# Supplementary material for: Characteristics of Pruritus in Various Clinical Variants of Psoriasis: Results of the Multinational, Multicenter, Cross-Sectional Study
Source: Life (Basel). 2021 Jun 27;11(7):623. doi: 10.3390/life11070623 (PMC8304988; doi:10.3390/life11070623)
Supplement: Supplementary file 1 [file life-11-00623-s001.zip › life-1263518-supplementary.pdf]

life

Article

Characteristics of Pruritus in Various Clinical Variants of Psoriasis: Results of the Multinational, Multicenter, Cross-Sectional Study

Kamila Jaworecka [1], Dominika Kwiatkowska [1], Luiza Marek [2], Funda Tamer [3], Aleksandra Stefaniak [4], Magdalena Szczegielniak [5], Joanna Chojnacka-Purpurowicz [6], Monika Matławska [6], Ayla Gulekon [3], Jacek C. Szepietowski [4], Joanna Narbutt [5], Agnieszka Owczarczyk-Saczonek [6] and Adam Reich [1,*]

[1] Department of Dermatology, Institute of Medical Sciences, Medical College of Rzeszow University, 35-055 Rzeszów, Poland; kamilajaworecka@gmail.com (K.J.); dominika.kwiatkowska.ur@gmail.com (D.K.)
[2] Department of Dermatology and Venerology, Faculty of Medicine, Ludwik Rydygier Collegium Medicum in Bydgoszcz, Nicolaus Copernicus University in Torun, 85-094 Bydgoszcz, Poland; Lui06@interia.pl
[3] Department of Dermatology, Gazi University School of Medicine, 06560 Ankara, Turkey; fundatmr@yahoo.com (F.T.); gulekona@gazi.edu.tr (A.G.)
[4] Department of Dermatology, Venerology and Allergology, Wroclaw Medical University,50-368 Wrocław, Poland; aleksandraannastefaniak@gmail.com (A.S.); jacek.szepietowski@umed.wroc.pl (J.C.S.)
[5] Department of Dermatology, Pediatric Dermatology and Oncology, Lodz Medical University, 91-347 Łódź, Poland; magda.szczegielniak@gmail.com (M.S.); joanna.narbutt@umed.lodz.pl (J.N.)
[6] Department and Clinic of Dermatology, Sexually Transmitted Diseases and Clinical Immunology, Faculty of Medicine, Collegium Medicum, University of Warmia and Mazury in Olsztyn, 10-959 Olsztyn, Poland; joannachojnacka2@wp.pl (J.C.-P.); mrs.matlawska@gmail.com (M.M.); aganek@wp.eu (A.O.-S.)
* Correspondence: adamandrzejreich@gmail.com

Citation: Jaworecka, K.; Kwiatkowska, D.; Marek, L.; Tamer, F.; Stefaniak, A.; Szczegielniak, M.; Chojnacka-Purpurowicz, J.; Matlawska, M.; Gulekon, A.; Szepietowski, J.C.; et al. Characteristics of Pruritus in Various Clinical Variants of Psoriasis: Results of the Multinational, Multicenter, Cross-Sectional Study. *Life* **2021**, *11*, 623. https://doi.org/10.3390/life11070623

Academic Editor: Kezhong Zhang

Received: 31 May 2021
Accepted: 24 June 2021
Published: 27 June 2021

Publisher's Note: MDPI stays neutral with regard to jurisdictional claims in published maps and institutional affiliations.

Copyright: © 2021 by the authors. Submitted for possible open access publication under the terms and conditions of the Creative Commons Attribution (CC BY) license (http://creativecommons.org/licenses/by/4.0/).

Supplementary Materials:

Table S1. Psoriasis severity and quality of life level of analyzed patients (data presented as means ± standard deviations; BSA - Body Surface Area, DLQI - Dermatology Life Quality Index, GPPSI - Generalized Pustular Psoriasis Severity Index, PASI - Psoriasis Area and Severity Index, PPSI - Palmoplantar Pustulosis Severity Index, sPGA - Static Physician Global Assessment).

	All Patients	Large-plaque Psoriasis	Nummular Psoriasis	Guttate Psoriasis	Palmoplantar Psoriasis	Psoriasis of the Scalp	Inverse Psoriasis	Erythrodermic Psoriasis	Palmoplantar Pustular Psoriasis	Generalized Pustular Psoriasis
PASI	13.2 ± 12.0	14.3 ± 9.8	11.1 ± 5.0	12.8 ± 9.2	4.4 ± 1.3	3.4 ± 3.7	5.9 ± 4.0	36.5 ± 9.9	-	-
BSA	22.2 ± 26.1	28.7 ± 21.4	19.9 ± 8.9	20.4 ± 10.0	5.6 ± 2.2	5.2 ± 4.9	6.2 ± 3.0	85.8 ± 21.8	2.8 ± 1.3	26.5 ± 17.2
sPGA	3.2 ± 1.0	3.2 ± 1.2	3.0 ± 0.9	3.0 ± 0.7	3.1 ± 0.7	2.8 ± 0.8	2.5 ± 0.9	4.1 ± 0.8	3.3 ± 0.9	3.4 ± 1.1
PPSI	-	-	-	-	-	-	-	-	7.0 ± 1.9	-
GPPSI	-	-	-	-	-	-	-	-	-	4.5 ± 1.8
DLQI	7.0 ± 1.9	11.9 ± 8.1	11.6 ± 6.8	10.9 ± 5.9	15.7 ± 9.5	9.4 ± 7.6	12.6 ± 7.5	17.9 ± 7.5	10.5 ± 7.8	15.5 ± 8.1

Table S2. Selected features and descriptions of pruritus (*p values according to χ^2 test).

	All Patients	Large-plaque Psoriasis	Nummular Psoriasis	Guttate Psoriasis	Palmoplantar Psoriasis	Psoriasis of the Scalp	Inverse Psoriasis	Erythrodermic Psoriasis	Palmo-plantar Pustular Psoriasis	Generalized Pustular Psoriasis	P*
Frequency of pruritus: n (%)											
- Everyday	113 (57.4)	25 (58.1)	13 (50)	14 (63.6)	7 (70)	10 (43.5)	4 (40)	13 (81.3)	23 (63.9)	4 (36.4)	0.66
- Few times a week	51 (25.9)	12 (27.9)	9 (34.6)	5 (22.7)	1 (10)	6 (26.1)	4 (40)	2 (12.5)	8 (22.2)	4 (36.4)	
- At least once a month	23 (11.7)	3 (7)	2 (7.7)	3 (13.6)	1 (10)	6 (26.1)	2 (20)	0 (0)	4 (11.1)	2 (18.2)	
- Less than once a month	10 (5)	3 (7)	2 (7.7)	0 (0)	1 (10)	1 (4.3)	0 (0)	1 (6.2)	1 (2.8)	1 (9.1)	
The most intense pruritus: n (%)	71 (36)	9 (20.9)	9 (34.6)	10 (45.5)	2 (20)	11 (47.8)	4 (40)	7 (43.8)	17 (47.2)	1 (9.1)	0.03
- When the skin lesions appear	59 (29.9)	15 (34.9)	9 (34.6)	8 (36.4)	6 (60)	4 (17.4)	5 (50)	2 (12.5)	7 (19.4)	4 (36.4)	
- When the skin lesions are fully developed	53 (26.9)	15 (34.9)	6 (23.1)	4 (18.2)	2 (20)	7 (30.4)	0 (0)	3 (18.7)	10 (27.8)	6 (54.5)	
- When the skin lesions extend their size	14 (7.1)	4 (9.3)	2 (7.7)	0 (0)	0 (0)	1 (4.3)	1 (10)	4 (25)	2 (5.6)	0 (0)	

- Not related to the development of skin lesions											
Pruritus relieves: n (%)											
- Shortly after introducing antipsoriatic therapy	35 (17.8)	10 (23.3)	6 (23.1)	6 (27.3)	0 (0)	5 (21.7)	1 (10)	3 (18.7)	3 (8.3)	1 (9.1)	
- After significant improvement of psoriasis	81 (41.1)	18 (41.9)	11 (42.3)	9 (40.9)	4 (40)	6 (26.1)	2 (20)	7 (43.8)	18 (50)	6 (54.5)	
- When lesions fully disappear	47 (23.9)	9 (20.9)	5 (19.2)	4 (18.2)	5 (50)	8 (34.8)	4 (40)	1 (6.2)	9 (25)	2 (18.2)	0.6
- Pruritus persists in spite of clearance of skin lesions	10 (5)	1 (2.3)	1 (3.8)	0 (0)	0 (0)	2 (8.7)	2 (20)	2 (12.5)	2 (5.6)	0 (0)	
- I do not know	24 (12.2)	5 (11.6)	3 (11.5)	3 (13.6)	1 (10)	2 (8.7)	1 (10)	3 (18.8)	4 (11.1)	2 (18.2)	
Descriptions of pruritus											
Pure itch: n (%)	62 (31.5)	14 (32.6)	8 (30.8)	10 (45.5)	2 (20)	11 (47.8)	1 (10)	4 (25)	8 (22.2)	4 (36.4)	0.16
Burning: n (%)	75 (38.1)	19 (44.2)	9 (34.6)	9 (40.9)	4 (40)	7 (30.4)	4 (40)	6 (37.5)	14 (38.9)	3 (27.3)	0.98
Tingling: n (%)	47 (23.9)	10 (23.3)	7 (26.9)	3 (13.6)	1 (10)	6 (26.1)	2 (20)	5 (31.3)	11 (30.6)	2 (18.2)	0.88
Painful itch: n (%)	44 (22.3)	9 (20.9)	3 (11.5)	3 (13.6)	5 (50)	2 (8.7)	2 (20)	2 (12.5)	15 (41.2)	3 (27.3)	<0.05
Pinching: n (%)	41 (20.8)	11 (25.6)	6 (23.1)	3 (13.6)	3 (30)	2 (8.7)	3 (30)	3 (18.7)	6 (16.7)	4 (36.4)	0.52
Deep itch: n (%)	35 (17.8)	6 (14)	0 (0)	0 (0)	3 (30)	2 (8.7)	2 (20)	3 (18.7)	14 (38.9)	1 (9.1)	<0.01
Point itch: n (%)	28 (14.2)	3 (7)	1 (3.8)	4 (18.2)	1 (10)	4 (17.4)	4 (40)	3 (18.7)	6 (16.7)	2 (18.2)	0.26
Warming feeling: n (%)	27 (13.7)	6 (14)	1 (3.8)	2 (9.1)	4 (40)	2 (8.7)	1 (10)	1 (6.2)	8 (22.2)	2 (18.2)	0.2
Like walking ants: n (%)	23 (11.7)	2 (4.7)	2 (7.7)	3 (13.6)	1 (10)	5 (21.7)	2 (20)	4 (25)	4 (11.1)	1 (9.1)	0.41
Stinging: n (%)	20 (10.2)	2 (4.7)	2 (7.7)	1 (4.5)	2 (20)	1 (4.3)	0 (0)	1 (6.2)	8 (22.2)	3 (27.3)	0.09
Like the prick of a pin: n (%)	18 (9.1)	4 (9.3)	3 (11.5)	0 (0)	2 (20)	1 (4.3)	0 (0)	2 (12.5)	4 (11.1)	2 (18.2)	0.55
Superficial itch: n (%)	17 (8.6)	6 (14)	3 (11.5)	2 (9.1)	1 (10)	0 (0)	1 (10)	2 (12.5)	2 (5.6)	0 (0)	0.64
Biting: n (%)	13 (6.6)	2 (4.7)	1 (3.8)	1 (4.5)	0 (0)	2 (8.7)	0 (0)	2 (12.5)	2 (5.6)	3 (27.3)	0.16
Like a spool and a needle: n (%)	10 (5.1)	2 (4.7)	2 (7.7)	0 (0)	1 (10)	2 (8.7)	0 (0)	2 (12.5)	0 (0)	1 (9.1)	0.48
Acute itch: n (%)	9 (4.6)	5 (11.6)	0 (0)	1 (4.5)	1 (10)	0 (0)	0 (0)	1 (6.2)	1 (2.8)	0 (0)	0.29
Drilling: n (%)	4 (2)	2 (4.7)	1 (3.8)	0 (0)	0 (0)	0 (0)	0 (0)	0 (0)	0 (0)	1 (9.1)	0.46

Like electric shock: n (%)	3 (1.5)	0 (0)	1 (3.8)	0 (0)	0 (0)	0 (0)	0 (0)	0 (0)	1 (2.8)	1 (9.1)	0.45
Feeling cold: n (%)	1 (0.5)	0 (0)	0 (0)	0 (0)	0 (0)	0 (0)	0 (0)	0 (0)	0 (0)	1 (9.1)	0.02
